# Power Generation Performance of a Pilot-Scale Reverse Electrodialysis Using Monovalent Selective Ion-Exchange Membranes

**DOI:** 10.3390/membranes11010027

**Published:** 2021-01-01

**Authors:** Soroush Mehdizadeh, Yuriko Kakihana, Takakazu Abo, Qingchun Yuan, Mitsuru Higa

**Affiliations:** 1 Aston Institute of Materials Research (AIMR), Aston University, Birmingham B4 7ET, UK; soroushm@aston.ac.uk (S.M.); yuanq@aston.ac.uk (Q.Y.); 2 Process Engineer Development, Qualitetch Component Ltd., March PE15 8QW, UK; 3 Blue Energy Center for SGE Technology (BEST), Yamaguchi University, 2-16-1 Tokiwadai, Ube, Yamaguchi 755-8611, Japan; kakihana@yamaguchi-u.ac.jp; 4 Graduate School of Sciences and Technology for Innovation, Yamaguchi University, 1677-1 Yoshida, Yamaguchi 753-8511, Japan; b020vf@yamaguchi-u.ac.jp

**Keywords:** RED, monovalent permselective membrane, RO brine, uphill effect

## Abstract

Reverse electrodialysis (RED) is a promising process for harvesting energy from the salinity gradient between two solutions without environmental impacts. Seawater (SW) and river water (RW) are considered the main RED feed solutions because of their good availability. In Okinawa Island (Japan), SW desalination via the reverse osmosis (RO) can be integrated with the RED process due to the production of a large amount of RO brine (concentrated SW, containing ~1 mol/dm^3^ of NaCl), which is usually discharged directly into the sea. In this study, a pilot-scale RED stack, with 299 cell pairs and 179.4 m^2^ of effective membrane area, was installed in the SW desalination plant. For the first time, asymmetric monovalent selective membranes with monovalent selective layer just at the side of the membranes were used as the ion exchange membranes (IEMs) inside the RED stack. Natural and model RO brines, as well as SW, were used as the high-concentrate feed solutions. RW, which was in fact surface water in this study and close to the desalination plant, was utilized as the low-concentrate feed solution. The power generation performance investigated by the current-voltage (I–V) test showed the maximum gross power density of 0.96 and 1.46 W/m^2^ respectively, when the natural and model RO brine/RW were used. These are a 50–60% improvement of the maximum gross power of 0.62 and 0.97 W/m^2^ generated from the natural and model SW, respectively. The approximate 50% more power generated from the model feed solutions can be assigned to the suppression of concentration polarization of the RED stack due to the absence of multivalent ions.

## 1. Introduction

Increasing world energy demand, especially in the last few decades, has caused the continuous use and burning of fossil fuels [1,2,3,4]. Consumption of more fossil fuels has increased concerns regarding different environmental aspects (e.g., global warming, air pollution, and CO_2_ emissions), which has resulted in more attention on new and renewable sources of energy [1]. Among the different sources of renewable energy (e.g., solar, wind, wave, geothermal, and biomass), salinity gradient energy (SGE) is a promising and sustainable resource [2]. In 1954, Pattel made a novel demonstration of SGE as an electrochemical potential between two solutions with different salinities [5]. In this regard, solvated ions in solutions have an electrochemical potential to move from a high concentrate to a low concentrate area until they reach equilibrium. The global salinity power by considering all discharge of river water (RW) into seawater (SW) has theoretically estimated approximately 1.4–2.6 TW of energy, which is a considerable amount of energy compared with world energy demand [6,7]. However, the lack of required equipment to convert SGE into an appropriate energy form was one of the constraints faced by Pattel when they presented their work on SGEs. In the last decade, different SGE-based processes have been introduced and improved mainly because of advancements in membrane technology [8,9,10]. Membrane-based reverse electrodialysis (RED) is a sustainable process that uses SGE for energy generation [1,2,3,4,5,6,7,8,11,12,13]. Developing RED can be an appropriate energy production method because it directly converts salinity power into electrical energy [14]. In RED, high and low concentrate solutions flow alternatively through stacked anion exchange membranes (AEMs) and cation exchange membranes (CEMs) [15]. Anions and cations migrate in opposite directions from high to low concentrate compartments (HCC and LCC) through stacked AEMs and CEMs, respectively. The internal ion transportation stack makes a potential difference through the RED stack and converts it into an external electric current using a suitable electrolyte and electrode system.

Operation conditions (e.g., feed flowrate, feed temperature, feed concentration) and stack scaling up in RED have always been considered as important research topics in the last two decades [14]. In this regard, SW and RW are well known as the most common feed solutions for the RED process because of their availability. Theoretically, 2.5 MJ of energy can be harvested by mixing 1 m^3^ of model RW (0.015 M NaCl) with a large amount of model SW (0.5 M NaCl) [16]. However, the actual energy obtained from SW and RW using RED is much lower because of different reasons such as fouling, low membrane permselectivity, membrane and solution resistance, and pressure drop [14]. The RED power output can increase from 0.05 W/m^2^_membrane_ at the beginning to approximately 1.02 W/m^2^_membrane_ by modifying the RED stack (e.g., improving electrode and electrolyte system, and decreasing the feed solution pressure drop by improving the hydrodynamic condition), changing operation conditions, and improving applied membranes when model SW and RW are used as feed solutions [8,17,18,19,20]. Previous studies have shown that increasing the RED feed salinity ratio has a significant effect on power output. Hence, using brine (5 M NaCl) instead of SW as a concentrate feed solution can be an effective way to increase power output [17]. In this regard, Daniilidis et al. reported a power density of 6.7 W/m^2^ using a RED stack with a 100 µm intermembrane distance using model brine and RW feed solutions [21].

In addition to brine, other types of natural solutions have been considered as RED feed solutions due to their accessibility, such as desalination brine, treated wastewater, and saline wastewater, [22]. However, when using natural feed solutions in RED, the presence of divalent ions (e.g., Mg^2+^, Ca^2+^, and SO_4_^2−^) together with NaCl in feed solutions showed a significant impact on RED performance [20,23,24]. For instance, the molar fraction of 10% MgSO_4_ with 90% NaCl in both RED feed solutions (SW and RW) represented an approximately 29% to 50% decrease in power density because of the uphill transport behavior of divalent ions against their concentration gradient, which leads to a decrease in the stack voltage and an increase in the membrane resistance [25]. Therefore, using monovalent ion-selective membranes is an option to prevent or reduce divalent ions’ undesired uphill transport [26,27]. In this regard, the impact of divalent ions on the RED power density was much lower than that of standard membranes [28]. However, by increasing the concentration of divalent ions in the feed solutions, the monovalent ion-selective membrane resistance again divalent ions desired downhill transport from HCC to LCC and reduced the RED performance. Therefore, we believe that using a one-side monovalent selective membrane with a selective layer facing a low-concentrate compartment (LCC) would be more efficient in preventing or reducing the undesired uphill transport effect.

In addition to collecting suitable membranes, enhancing RED is recognized as a necessary approach to upgrade from lab-scale to commercialization of the RED process. To enhance RED, Veerman et al. investigated the effect of residence time on power density and compared the RED performance with co-current and counter-current for a 50-cell RED stack with a 18.75 m^2^ membrane effective area [18]. The maximum power density of approximately 0.63 W/m^2^ was reported using model SW and RW in the co-current state. In addition, Tedesco et al. reported the data of three RED pilot-scale stacks performed by *REAPower* with a total of a 400 m^2^ membrane effective area using brine from salt plants and brackish water (0.03 M NaCl) to reach 1 KW power density [29,30]. However, they obtained 700 and 330 W using model and natural feed solutions respectively, because of the effect of the non-NaCl substance in natural feed solutions. Although the salinity ratio between HCC and LCC was substantially high, the maximum power density of 0.8 W/m^2^ was reported for stack-3 (194 m^2^ membrane effective area) using natural feed solutions, which appears to be lower than expected. The low power density of stack-3 is due to the presence of the non-NaCl substance in feed solutions, and might be related to the design and hydrodynamic conditions of the stack. Although some studies have been conducted on enhancing the RED process, they are insufficient, and we still believe that there are different unknown phenomena in the RED pilot-scale.

Yasukawa et al. evaluated the steady-state power generation of a bench-scale RED stack (40 m^2^ total membrane effective area) using RO brine from the SW desalination plant and discharge from the sewage treatment plant, and reported an energy efficiency of 17–26% [31]. Co-locating a reverse osmosis (RO) SW desalination plant with the RED process can be an interesting opportunity for energy generation together with water desalination [32]. Using RO brine because of its potential as a concentrate feed solution for the RED process instead of just purging it into the SW is beneficial for fully or partially recovering energy from desalination. In this study, we evaluated the performance of the RED pilot plant (RED stack) with 299 cell pairs and a 179.4 m^2^ membrane effective area. Both the model and the natural SW/RW as well as the RO brine/RW feed solutions combination were applied in this study. Because the RO brine passed different filtration stages in the SW desalination plant, it required lower energy consumption for pretreatment and filtration before pumping into the RED stack. RW was considered instead of wastewater in this study as a RED feed solution. In addition, this RED stack is equipped with one-side monovalent selective membranes with a selective layer facing the LCC to diminish the effect of uphill transport for the first time in the pilot scale. Both current–voltage (I–V) and constant current (CC) tests were performed at different feed flow rates to determine the maximum power density and effect of concentration polarization, respectively.

## 2. Case Study

### 2.1. Desalination Unit

**Figure 1:**
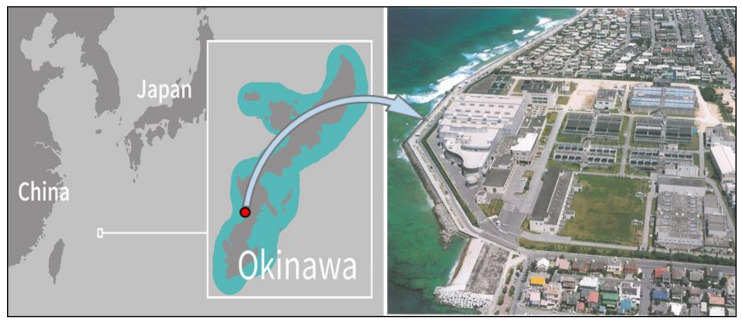
Water desalination plant of Okinawa islands in Chatan town, Japan [33].

The Okinawa SW desalination plant was constructed in Chatan town, Okinawa island, Japan, in early 1996, with an area of approximately 12,000 m^2^, as shown in Figure 1. This plant has a recovery rate of approximately 40% and applies the RO method using a spiral-type aromatic polyamide membrane to make freshwater from SW. The maximum capacity of freshwater production is approximately 40,000 m^3^/day, while the RO brine, which is the concentrated SW from the RO process, discharges into the sea with a flow rate of 60,000 m^3^/day at the maximum production rate. We used surface water, which is a mixture of water from the river and dam, for the low-salinity solution. For simplicity, here, we refer to the water as RW. Table 1 shows the ion composition of the SW, RO brine, and surface water (RW). This plant showed an appropriate potential for energy harvesting using the RED process because of the availability of SW and the high amount of RO brine.

### 2.2. RED Pilot Plant

#### 2.2.1. Feed Solution

**Figure 2:**
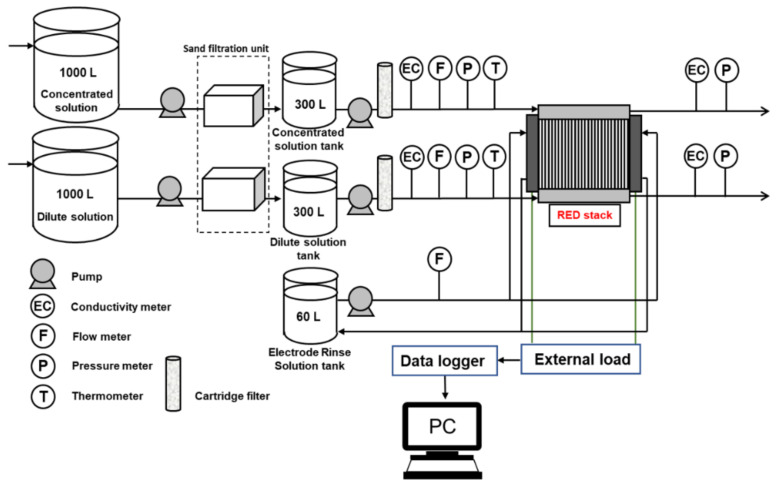
Reverse electrodialysis (RED) plant layout includes RED stack, pre-treatment system, and pumping system.

Natural RW, SW, and RO brine were connected by intake lines to the RED pilot plant and were used as low-concentrate and high-concentrate RED feed solutions. The RED plant layout is shown in Figure 2. Two storage tanks (1 m^3^ capacity) were used to save the feed solution if the flow of the feed suddenly disconnected because of unexpected issues. In addition, two more tanks with a volume of 300 L were used for natural feed solution storage and model feed solutions. In contrast to most of the literature on the subject, model feed solutions were prepared based on the ion concentration (not conductivity) of natural feed solutions because multivalent ions also affect solution conductivity. In our previous study, we used RO brine from Mamizu Pia (Fukuoka, Japan), and its conductivity was approximately 90 mS/cm [31]. Therefore, we set the conductivities of the RO brine (~1 mol/dm^3^ NaCl) and SW (~0.53 mol/dm^3^ NaCl) as 90 ± 1 mS/cm and 50 ± 1 mS/cm, respectively.

#### 2.2.2. Pre-Treatment

Natural feed solutions were first fed into AF-4 type (ZEOLITE Co., Ltd., Fukuoka, Japan) sand filtration with 1.26 m^3^/h filtration capacity. Two tanks with 300 L capacity were used to store the natural feed solution. In addition, all feed solutions were passed through a 0.45 µm cartridge filter before being fed into the RED stack using two CM1-3 (GRUNDFOS Pump Co., Ltd., Hamamatsu, Japan) pumps.

#### 2.2.3. RED Stack

The RED stack with 299 cell pairs and 179.4 m^2^ total membrane effective area was installed in the SW desalination unit. One side monovalent selective Neosepta^®^ CIMS and Neosepta^®^ ACS-8T (ASTOM. Corp, Tokyo, Japan) as CEM and AEM respectively, were stacked alternatively in the RED stack with a selective layer facing the LCC. Table 2 shows the properties of CIMS and ACS-8T measured in the lab, and the details are shown in the Appendix A. Feed solutions flow co-current from the bottom of the stack to the top to cover all the membrane effective area. Two Pt electrodes were used as the cathode and anode at the two ends of the RED stack. In addition, Na_2_SO_4_ solution was used as the electrolyte with a conductivity of 50 ± 2 mS/cm and fed into the RED stack using a MX-70VM32 magnet drive pump (IWAKI CO., Ltd., Tokyo, Japan). The electrolyte flow rate was changed by changing the feed solutions flow rate to keep the pressure difference in balance between feed and electrolyte compartments. In addition, 200 µm woven spacers were used to maintain the distance between the membranes equipped with gaskets to prevent leakage. The inlet feed solutions flow rate was measured using FD-P20 (KEYENCE CORPORATION, Osaka, Japan) flow sensors. The pressure and temperature of the inlet and outlet solutions were measured using FHXI-200KP-02-V (OPTEX FA Co., Ltd., Kyoto, Japan) and V1-2000-R3/8CF-M3Y (NIHONDENSOKU Co., Ltd., Osaka, Japan), respectively. In addition, the conductivities of both the inlet and outlet solutions were measured using an EC-430 (SUNTEX Instruments Co., Ltd., Taipei, Taiwan) conductive meter. All data were recorded using a GT SoftGOT2000 (Mitsubishi Electric Corporation, Tokyo, Japan) logger.

## 3. Experimental Procedure

### 3.1. RED Performance Test

The RED stack was tested under both current-voltage (I–V) and constant current (CC) conditions at different feed flow rates. Both I–V and CC conditions were established using PLZ664WA (KIKUSUI electronics corporation, Japan) multifunctional DC electronic load and recorded using a logger. In the case of I–V tests, the current increased from zero by a sequence of 10 mA/s until the voltage became zero. CC tests were performed by measuring the power output of the RED stack at a fixed current value until the power becomes stable. In both types of tests, the feed solution flow rates increased to investigate the effect of the feed flow rate on the RED stack power generation. The number of performance tests also depended on the feed solution availability provided by the water desalination unit.

### 3.2. Open Circuit Voltage (OCV)

The maximum voltage of the RED stack under the zero-current condition is known as the open-circuit voltage (OCV), which shows the potential of the RED stack for power production. The OCV of the RED stack during all RED tests at different feed flow rates was recorded. In addition to the actual voltage, the theoretical OCV with the assumption of NaCl as the main component in solutions were calculated using the Nernst equation, as follows:

(1) $$OCV_{stack}=N_{cell}.\alpha \;\frac{R.T}{F}\ln\frac{\gamma_{H}C_{H}}{\gamma_{L}C_{L}}$$

where *N_cell_* and *α* are the number of RED stack cell pairs and the average permselectivity of the CEM and AEM (-), respectively. *R* is the gas constant (8.314 J·mol^−1^·K^−1^), *T* is the temperature (K), and *F* is the Faraday constant (96,485 C/mol). γ and *C* are the average ion (Na^+^ and Cl^−^) activity coefficient (-) and ion concentration (mol/dm^3^), respectively. Subscripts *L* and *H* refer to the low-and high-concentrated solutions. To investigate the effect of ion diffusion through OCV measurements, the theoretical OCV of the RED stack at both the inlet and outlet of the feed solutions were calculated. The activity coefficient of NaCl solutions at different concentrations was calculated using solution conductivity. The respective methods and equations are shown in the Appendix B.

### 3.3. Stack Resistance

The internal resistance of the RED stack consists of ohmic and non-ohmic resistance (e.g., concentration polarization, change of bulk solution concentration) [13]. The ohmic regime of RED stack resistance, which is called theoretical resistance, contains the sum of the solution compartments and membrane resistances, as follows:

(2) $$R_{Ohmic}=N_{cells}(\beta_{sol}(R_{H}+R_{L})+\beta_{mem}\left(R_{AEM}+R_{CEM}\right))$$

where $N_{cell}$ is the number of cell pairs. *R_H_* and *R_L_* are the resistances of the high concentrate and low concentrate compartments, respectively. In addition, *R_AEM_* is AEM resistance, *R_CEM_* is CEM resistance, $\beta_{sol}$ is the spacer shadow effect on solution compartments, and $\beta_{mem}$ is the spacer shadow effect on membrane resistance [13]. However, the actual resistance of the RED stack containing ohmic and non-ohmic regimes was obtained using the slope of the I–V curves and Ohm’s law, as follows: *E_stack_* and *R_stack_* are the voltage and resistance of the RED stack, respectively:

(3) $$E_{stack}=OCV-R_{stack}I$$

### 3.4. Gross Power Output

The RED stack gross power output, *P_gross_*, was calculated by multiplying the stack voltage (*E_stack_*) by the current, as shown by Equation (4). In addition, the net power ($P_{net}$) was calculated by subtracting the pumping energy due to hydraulic losses from the power output shown by Equation (5), as follow:

(4) $$P_{gross}=E_{stack}.I$$

(5) $$P_{net}=P_{gross}-\frac{\Delta p_{L}Q_{L}+\Delta p_{H}Q_{H}}{\eta_{pump}}$$

where Δp is the pressure drop, Q is the feed flow rate, and $\eta_{pump}$ is the pump efficiency (~85%). Gross power and net power density were evaluated by dividing the power and net power to the total membrane effective area, *A_total_*, as follows:

(6) $$P_{d,gross}=\frac{P_{gross}}{A_{total}}$$

(7) $$P_{d,net}=\frac{P_{net}}{A_{total}}$$

## 4. Results and Discussion

### 4.1. Open Circuit Voltage (OCV)

**Figure 3:**
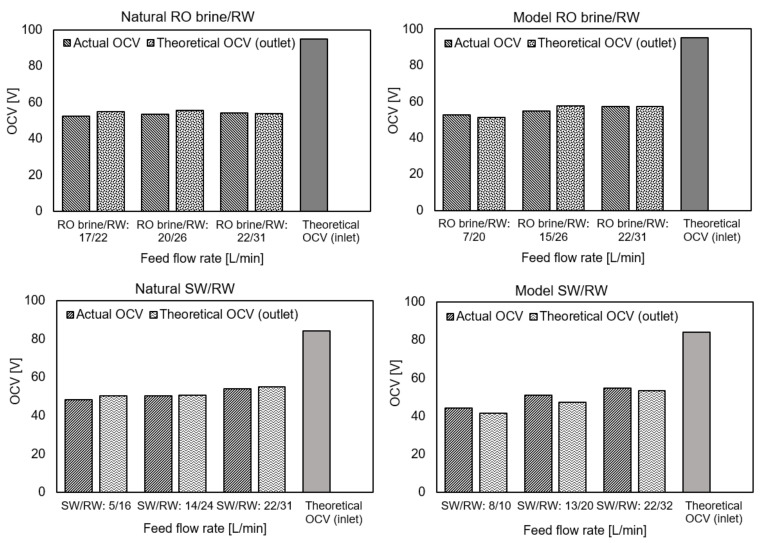
Actual and theoretical open circuit voltage (OCV) using natural and model feed solution.

Among all RED performance measurements using natural and model feed solutions, three actual OCVs were collected for each type of feed solution combination based on increasing the feed flow rate, as shown in Figure 3. In addition, because the conductivity of the feed solutions was recorded at both the inlet and outlet during the OCV measurement (zero current), the theoretical OCVs were calculated through the inlet and outlet feed solutions using the Nernst equation, respectively. In the case of natural solutions, the activity coefficients and concentrations were calculated using the model solution (aqueous NaCl) equations. The conductivity of the feed solutions at the inlet and outlet at the zero-current condition and all OCVs are shown in Appendix C. Through theoretical calculation, the permselectivity of membranes is assumed to be 100% for simplification. In all cases, the actual OCV was increased by increasing the feed flow rates. The salinity ratio decreased by passing the feed solutions through the RED stack compartments because of ion diffusion from the high-concentrate to low-concentrate compartments. Ion diffusion occurs because of a high salinity ratio and an unideal membrane permselectivity number, which is lower than 100% in a real case. This would allow co-ions to pass through the membranes together with counter-ions and reduce the salinity ratio and the respective OCV. In this regard, increasing the feed flow rates makes the salinity ratio less affected by ion diffusion because of the lower residence time for feed solutions to pass the RED stack. In addition, the OCV difference of the RED stack when using natural and model feed solutions was approximately 3% and significantly lower than that of the literature, which was approximately 10–15% [20,22,25,26,28,29,30,34,35]. In fact, facing the monovalent selective layer of membranes towards a low concentrate compartment can effectively decrease the uphill transport following up with divalent ion diffusion, which significantly affected the RED stack OCV.

Under the highest feed flow rate conditions, the actual OCV of the RED stack using the model and natural RO brine/RW as well as SW/RW feed solutions were approximately 60% and 65% of the theoretical inlet OCV, respectively. This means that 35–40% of OCV or RED stack potential was wasted, and it is clearer in the pilot-scale RED stack. The higher OCV_out_/OCV_in_ ratio using SW/RW compared with RO brine/RW feed combination was because of the lower ion diffusion in the SW/RW feed configuration because of the lower salinity ratio compared with RO brine/RW. In addition, the actual permselectivity of the membranes decreased with increasing feed solution concentrations. Therefore, a lower concentration of SW compared with RO brine leads to higher permselectivity of membranes in the case of using SW/RW and lower co-ion diffusion through the membranes. In all cases, the OCVs were obtained almost the same as the theoretical outlet OCV, which indicated that the ion diffusion occurred rapidly at the beginning when feed solutions flowed into compartments, and the salinity ratio among the rest of the compartments was almost the same as the outlet feed solutions.

### 4.2. Stack Resistance (Ω)

**Figure 4:**
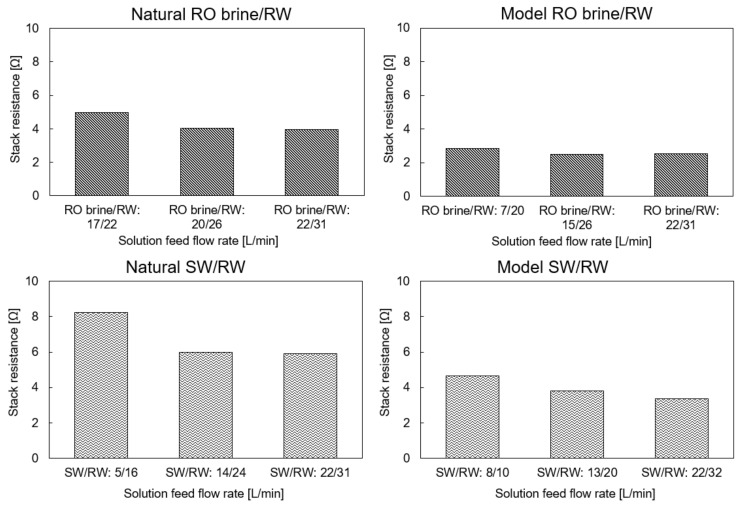
RED stack resistance using the natural and model feed solution with different flow rates.

The stack resistance using the model and natural feed solutions in three different flow rate configurations is shown in Figure 4. Increasing the feed flow rate would improve ion distribution, decrease the concentration polarization effect, and decrease the stack resistance. Therefore, in all cases, the stack resistance decreased with an increase in the feed flow rate. In addition, because RO brine has higher conductivity than SW, the RED stack resistance using the natural and model RO brine/RW was approximately 35–45% lower than that using the natural and model SW/RW, respectively. Generally, the RED stack showed the same range of resistance as the Tedesco et al. project using the RED pilot-scale with 194 m^2^ membrane effective area using concentrated brine (215 mS/cm) and brackish water (0.9 mS/cm) [29]. In this study, the stack resistance was 2.2–3.5 Ω when using model feed solutions and 4–3.3 Ω when using natural feed solutions as maximum flow rates (26–32 L/min). It is worth noting that the concentration of feed solutions in their project was much higher than in our study, which indicated that our RED stack configuration showed better performance.

As mentioned, multivalent ions lead to an increase in membrane resistance because of their higher hydride radius and valence than monovalent ions, which makes them attach stronger to membrane charged groups in the membrane bulk and make ion transportation difficult. Here, the difference between RED stack resistance using natural and model feed solutions directly indicates the effect of multivalent ions, which is in natural feed solution.

### 4.3. RED Performance Using the Natural RO Brine and RW

**Figure 5:**
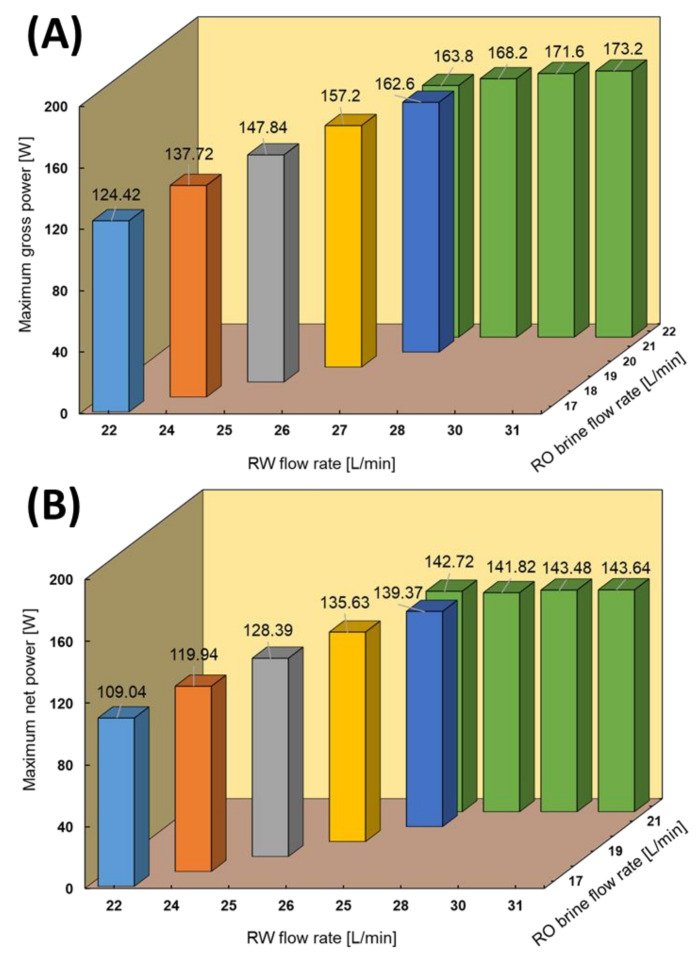
RED stack performance using the natural RO brine/RW feed solutions. (**A**) Maximum gross power, (**B**) Maximum net power.

Different feed flow rates of the natural RO brine and RW were applied as RED feed solutions to investigate the effect of feed flow rates on power and obtain maximum power output. Increasing the feed flow rate could significantly impact the RED power output due to an increase in the ion distribution, keeping the salinity ratio constant, and decreasing the concentration polarization. In this case, the RW and RO brine flow velocities increased from 1.25 to 1.73 cm/s and 0.95 to 1.17 cm/s, respectively. The RO brine flow rate increased by increasing the RW flow rate to maintain the pressure balance through HCC/LCC and minimize solution leakage. Figure 5 shows the maximum gross and net power output of the RED stack obtained at different feed flow rates through the I–V test condition. The maximum power output increased from 124.42 (0.69 W/m^2^) to 173.2 W (0.96 W/m^2^) by increasing the RW flow rate from 22 to 31 L/min and increasing the RO brine from 17 to 22 L/min. To the best of our knowledge, the gross power density of 0.96 W/m^2^ is the maximum value obtained and reported among all pilot-scale RED stacks in the literature, which was approximately 0.38–0.84 W/m^2^ using different natural feed solutions [29,30,35]. Applying one side monovalent selective membrane significantly decreased the impact of uphill transport of divalent ions from low- to high-concentrate compartments [23]. In this case, the concentration of divalent ions in LCC is extremely low; therefore, the selective layer could effectively act as a barrier wall against uphill transport on the membrane surface.

The net power of the RED stack was calculated by subtracting the pumping energy, which is related to the feed flow rates, pressure drop, and pump efficiency from the gross power output. The recorded pressure drop of the RED stack under different conditions is shown in the Appendix C. Notably, the pumping energy consumption increases with an increase in the feed flow rate. Hence, the maximum net power of the RED stack can be defined as a trade-off power through gross power and pumping energy. In this case, although the pumping energy increased from 15 to 27.96 W by increasing the feed flow rates, the maximum net power of 143.64 W (0.80 W/cm^2^) was still obtained in the highest feed flow rate condition. To the best of our knowledge, this is the highest reported net power using natural feed solution on a pilot-scale compared with the literature, which was approximately 75 W in the maximum case [30].

**Figure 6:**
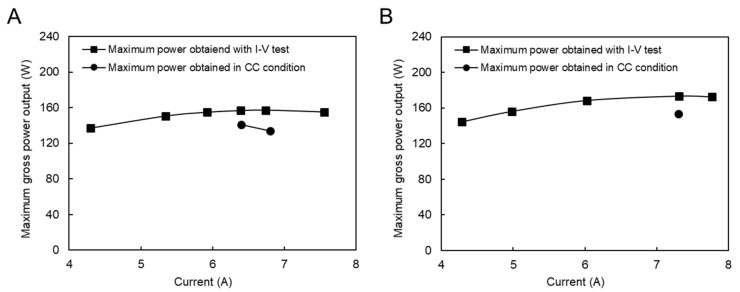
Constant current and I–V measurement with the natural RO brine and RW feed solution, (**A**) RO brine/RW; 26/20 (L/min), (**B**) RO brine/RW; 31/22 (L/min).

In addition, RED tests in constant current (CC) mode were performed to investigate the stability of power production and the effect of concentration polarization. The currents used were set around the maximum current power obtained by the I–V test. However, due to the feed flow rate limitation of the natural RO brine solution, only two feed flow rate conditions were examined, as shown in Figure 6. The difference between the maximum power obtained during I–V and CC conditions was due to the concentration polarization effect, which is higher in the CC condition and the stabilizing feed solution compartment concentrations. Because increasing the feed flow velocity diminishes the impact of concentration polarization, the difference in the maximum power obtained between I–V and CC modes decreased from 12.9% in Figure 6A to 10% in Figure 6B.

### 4.4. Performance with Model RO Brine and RW

**Figure 7:**
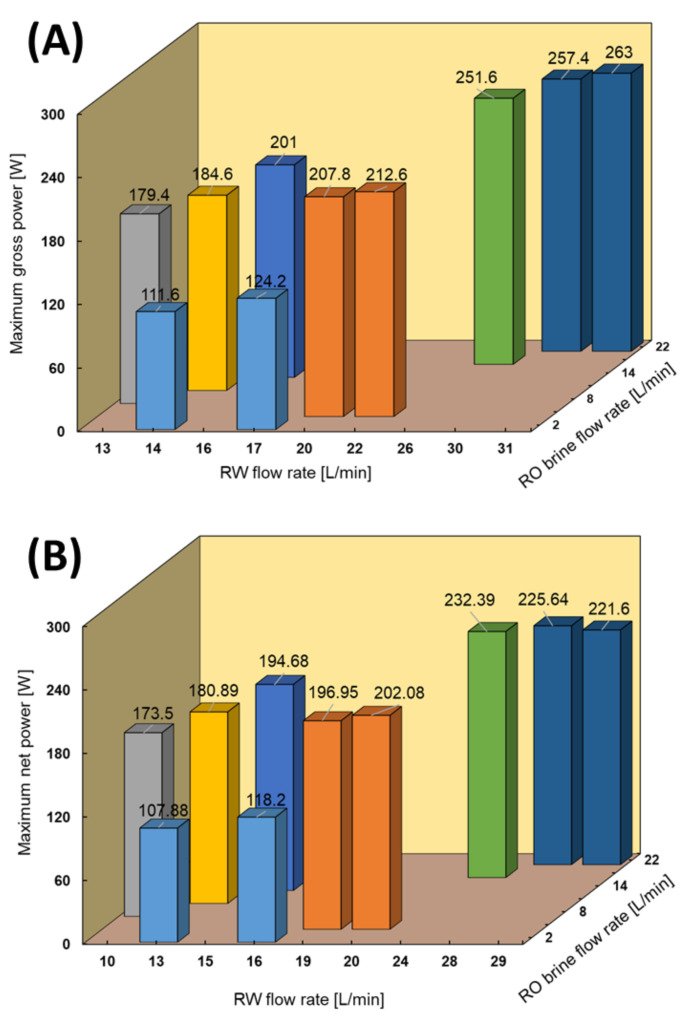
RED stack performance using model RO brine/RW feed solutions. (**A**) Maximum gross power density against feed flow rate, (**B**) Maximum net power against feed flow rate.

RED tests with model RO brine and RW were performed to investigate the effect of divalent ions on the performance. Since the RED stack is equipped with sand pre-filtration and a cartridge filter, we assumed that most of the natural organic materials that could significantly affect the RED performance were removed from the feed solutions [22]. In this case, except for a few tests at a high feed flow rate to obtain the maximum power generation, most of the measurements were performed using low feed flow rates due to the volume limitation of the model solution tank, as shown in Figure 7. As expected, the RED power out increased by increasing the feed low rates. For instance, the maximum power output increased by 5.5 W by increasing 1 L/min of the RW flow rate at a constant RO brine flow rate. The maximum gross power density reached 1.46 W/m^2^ (263 W), which is a significant amount compared with other studies by considering the salinity ratio of feed solutions. For instance, Tedesco et al. reported a maximum gross power density of 1.65 W/m^2^ using saline water (0.9 mS/cm) and concentrated brine (215 mS/cm), which has a salinity ratio approximately 1.4 times higher than that in our study [30].

The maximum obtained power output using model RO brine and RW was approximately 35% higher than the same conditions as the natural feed solutions. The impact of divalent ions in the natural feed solution increased the membrane resistance and higher conductivity of the model RO brine (90 mS/cm) than the natural RO brine (75 mS/cm) were the main reasons for obtaining higher power output.

The pumping energy increased from 3.7 to 31 W by increasing the feed flow rates, except for the highest feed flow rates (RO brine/RW: 22/31 L/min), which consumed 41.4 W. The latter was due to the significant increase in pressure drop of approximately 80 kPa in both high- and low-concentrate compartments. The pressure drop information is shown in the Appendix C. The maximum net power of 232.39 W (~1.29 W/m^2^) was obtained using RO brine/RW: 15/26 L/min because of the trade-off between the effect of feed flow rate on gross power and pumping energy.

**Figure 8:**
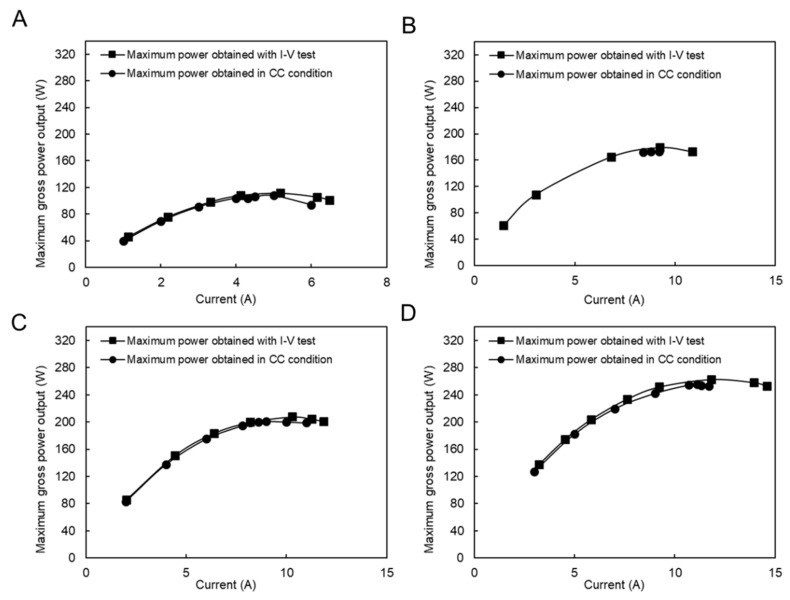
Constant current and I–V measurement with the model RO brine and RW feed solution, (**A**) RO brine/RW; 2/14 (L/min), (**B**) RO brine/RW; 8/13 (L/min), (**C**) RO brine/RW; 7/20 (L/min), (**D**) RO brine/RW; 22/31 (L/min).

In this case, the CC mode tests were performed under four different flow rate conditions, as shown in Figure 8. The applied currents were chosen around the maximum power output conditions during the I–V test condition. The difference between the maximum performance during the I–V test condition is reduced from 3% to 0.5% by increasing the feed flow rate, which is generally considerably lower than the same condition using a natural feed solution. In fact, the presence of divalent ions in natural feed solutions increased the concentration polarization impact because they had a higher valence, and their concentration increased around the membrane surface. In addition, their higher hydrate radius resists ion transportation, which increases the resistance and decreases the power output.

### 4.5. RED Performance with Natural SW and RW

**Figure 9:**
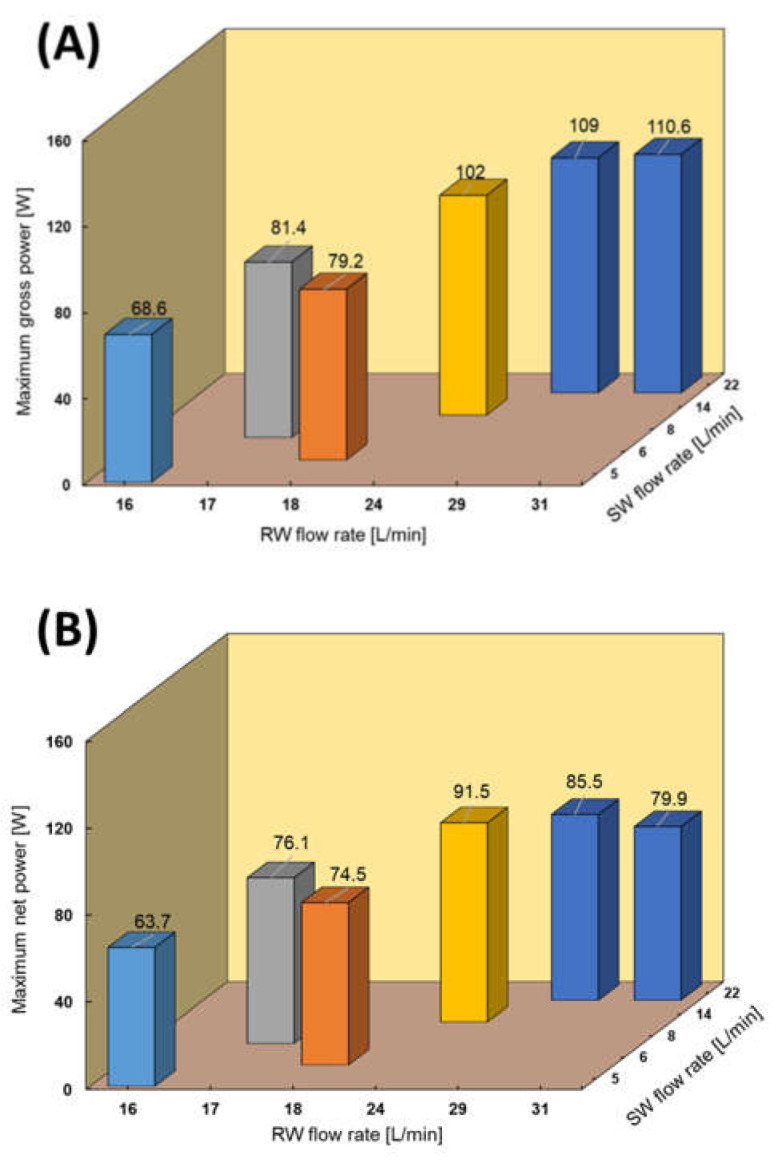
RED stack performance using natural SW/RW feed solutions. (**A**) Maximum gross power density against feed flow rate, (**B**) Maximum net power against feed flow rate.

RED tests using SW and RW were performed due to the availability of SW close to the RED stack and for comparing the performance with RED performance using RO brine as the feed solution. The RED stack performance using different flow rates of RW and SW is shown in Figure 9. The maximum power output increased from 68.6 (0.38 W/m^2^) to 110.6 W (0.62 W/m^2^) by increasing both feed flow rates. In this case, the maximum obtained gross power density was even higher than the reported RED performance with the same feed configuration in the lab-scale [20,22,23]. The RED power output increased faster by increasing the RW flow rate than by increasing the SW flow rate because of the significant effect of low concentrate compartment conductivity. In fact, increasing the RW flow rate keeps the conductivity of LCC at a lower value by refreshing the feed solution faster and maintaining a higher salinity ratio. The maximum obtained power decreased by approximately 35% compared with the RED performance using the natural RO brine/RW because of the decrease in the salinity ratio between feed solutions. As observed earlier, the pumping energy consumption increased from 4.7 to 30.7 W by increasing the feed flow rate, which was unexpectedly slightly higher than that under the same conditions using the natural RO brine/RW feed solutions. This can be because of the higher natural organic material that exists in SW compared with RO brine. RO brine has a higher concentration than SW and is supposed to cause a greater pressure drop in the RED stack channel. However, in this study, RO brine was pre-treated and filtered three times before being used in the RED process, including pre-treatment before being fed into the RO process, during the RO process by the UF membrane, and finally sand and cartridge filtration before the RED process. In contrast, the SW applied in the RED process just passed sand and cartridge filtration before the RED process. Therefore, the amount of natural organic materials in the SW must be higher than that in the RO brine, causing more pressure drop and fouling. The maximum net power was obtained using SW/RW: 14/24 L/min with a value of 91.5 W (0.51 W/m^2^).

**Figure 10:**
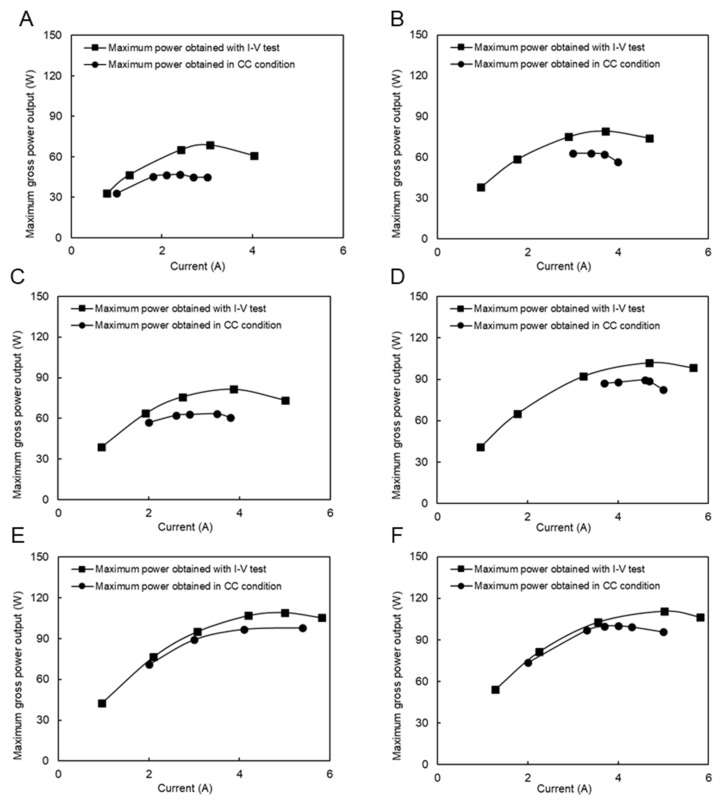
Constant current and I–V measurement with natural SW and RW feed solutions, (**A**) SW/RW; 5/16 (L/min), (**B**) SW/RW; 6/18 (L/min), (**C**) SW/RW; 8/17 (L/min), (**D**) SW/RW; 14/24 (L/min), (**E**) SW/RW; 22/29 (L/min), (**F**) SW/RW; 22/31 (L/min).

The RED tests in the CC condition were performed in six feed flow rate conditions, as shown in Figure 10. The difference between the maximum power obtained by the I–V measurement and the maximum power obtained in the CC mode decreased from 31% (Figure 10A) to 10% (Figure 10F) by increasing the feed flow rate due to a decrease in the polarization effect. These differences were higher than those when using natural RO brine and RW as feed solutions due to more filtration and pre-treatment steps on RO brine, as previously mentioned.

### 4.6. RED Performance with Model SW and RW

**Figure 11:**
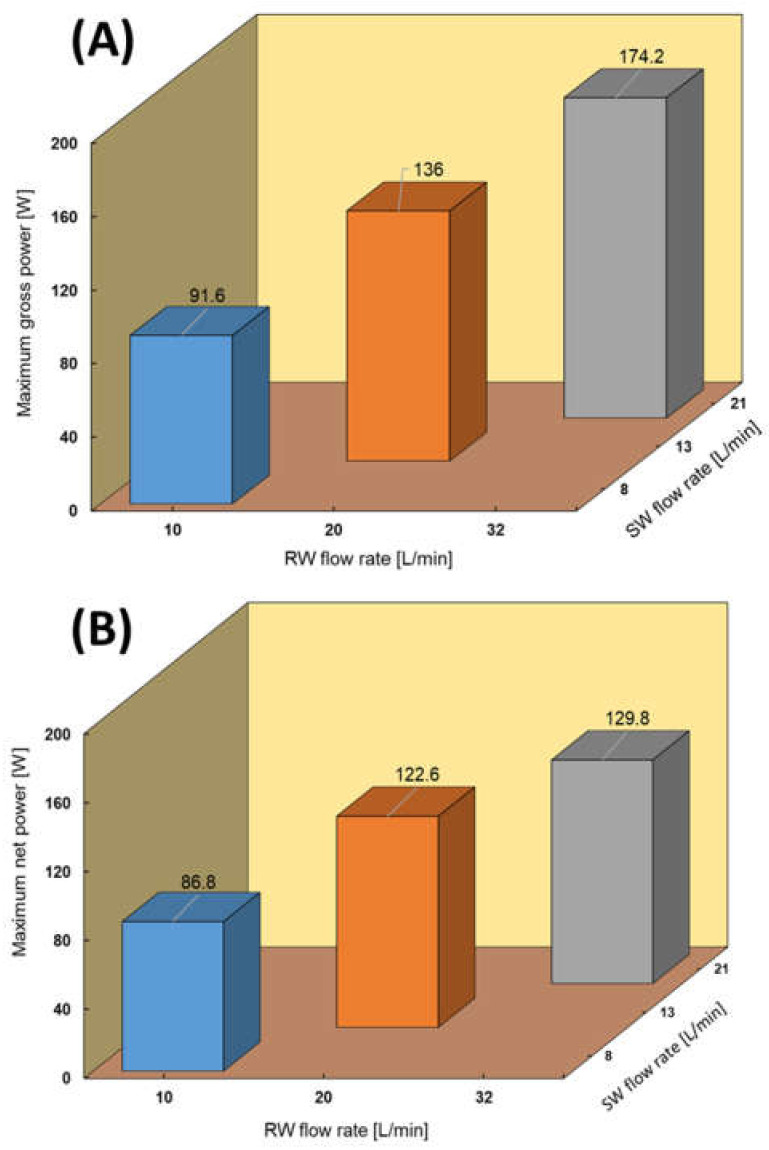
RED stack performance using model SW/RW feed solutions. (**A**) Maximum gross power density against feed flow rate, (**B**) Maximum net power against feed flow rate.

**Figure 12:**
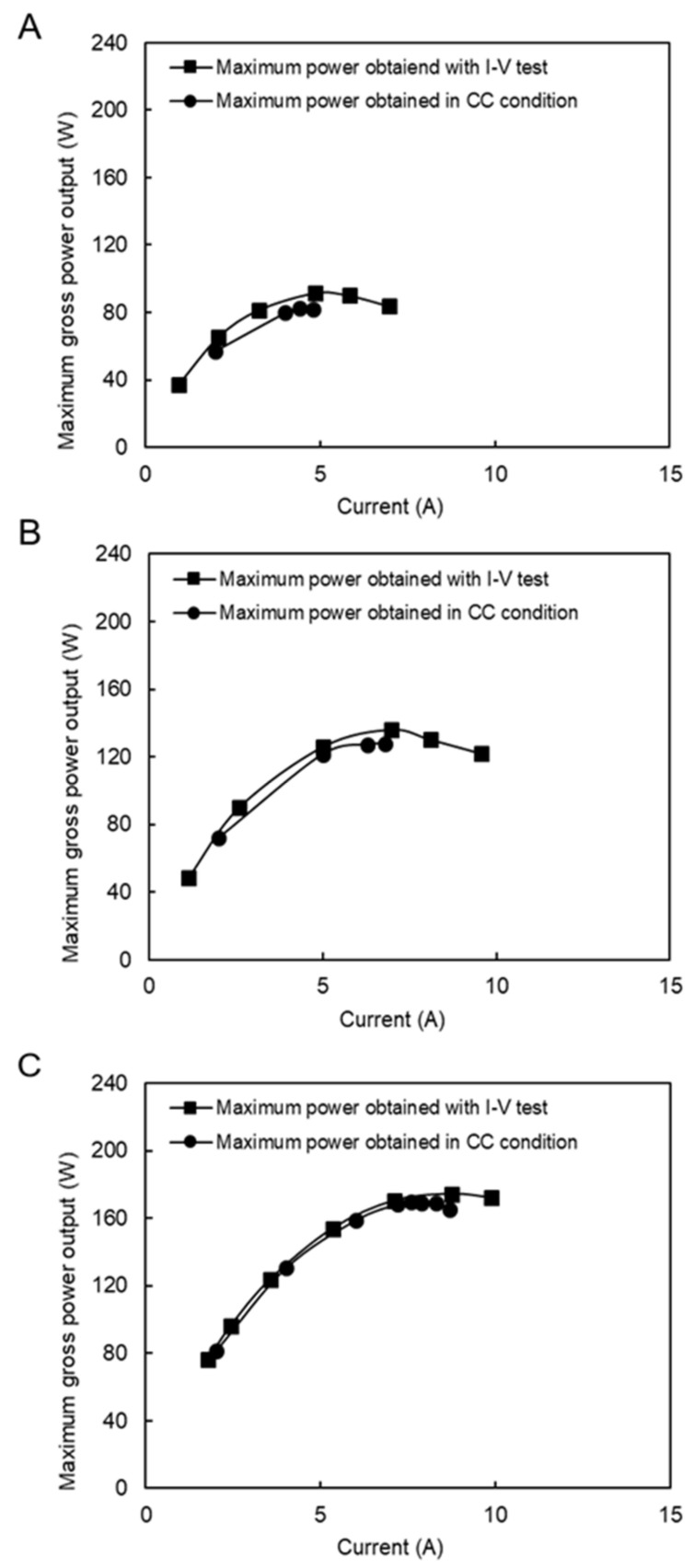
Constant current and I–V measurement with model SW and RW feed solutions (**A**) SW/RW; 8/10 (L/min), (**B**) SW/RW; 13/20 (L/min), (**C**) SW/RW; 21/32 (L/min).

RED tests using model SW and RW were performed at three different feed flow rate configurations through I–V and CC modes, as shown in Figure 11 and Figure 12, respectively. The maximum power output reached 174.2 W (0.97 W/m^2^), which is 37% higher than that obtained using natural SW and RW feed solutions. In addition, this difference was higher than that compared with the RED performance using the model and natural RO brine and RW, which was 35%. As previously mentioned, this is because the RO brine passes a 3-step pretreatment while natural SW only passes cartridge filtration. The pumping energy calculated was 4.8 and 13.4 W using SW/RW: 8/10 and 13/20 L/min feed flow rate, while it significantly increased to 44.4 W when using the highest feed flow rate condition (SW/RW: 21/29 L/min). The same jumping of pumping energy was also observed when using model RO brine/RW: 22/32 L/min feed solution flow rates. It seems that these feed flow rates are critical for the RED stack and cause pressure drop.

Figure 12A–C compares the maximum power output between the CC and I–V mode tests. In all cases, the maximum power obtained in the CC mode was close to the maximum power condition in the I–V tests. The difference decreased by increasing the flow rates from 2.7% to 10%. These values were much lower than the same situation with natural SW and RW because of divalent ions and natural organic materials in natural feed solutions.

### 4.7. Available Energy in Okinawa Water Desalination Plant

Improving the RED process scale in the pilot-scale can be an effective step for the commercialization of this process. However, the commercialization should be performed in a place with economic justification. Therefore, by considering the results of the pilot-scale experiments, we can provide an appropriate estimation for the need for improvements in the RED process on a commercial scale. In this study, the SW desalination plant in Okinawa, Japan, had a 60,000 m^3^/day RO brine production capacity. By considering the maximum power production condition (natural RO brine/RW: 22/31) and pumping energy, approximately 437 KW/day power can be produced. Therefore, approximately 900 m^2^ of solar panels with 18.7% efficiency will be required to produce this amount of energy. However, solar panels are affected by whether conditions (sunny or cloudy) and energy produced by them is limited by time (day and night), which makes this energy unstable.

## 5. Conclusions

The power generation performance of a pilot-scale RED stack, located at Okinawa (Japan) SW desalination plant, by the RO process was presented in this study. The RED stack consisted of 299 cell pairs with one side monovalent selective membrane with a selective layer facing a low concentrate compartment and a total of 179.4 m^2^ of the membrane effective area. SW and RO brine (concentrated SW) supplied from a desalination plant were used as concentrate feed solutions. RW was considered a low concentration feed solution. The maximum gross power output of 171.6 (0.96 W/m^2^) and 263 W (1.46 W/m^2^) were generated using the natural and model RO brine/RW feed solutions, respectively. The power generation decreased by approximately 34% when using the natural RO brine/RW compared with the model feed solutions because of the presence of divalent ions in the natural solution. In addition, the RED stack produced the maximum gross power of 110.6 (0.62 W/m^2^) and 174.2 W (0.97 W/m^2^) using natural and model SW/RW respectively, where the difference in the RED stack performance was approximately 36% because of the presence of multivalent ions in the natural feed solutions. The RED performance difference between natural and model feed solutions was observed to be lower than that reported in the literature because of the application of one-side monovalent selective membranes, which reduced the uphill transport.

In addition, the RED performance was evaluated under constant current (CC) conditions. In the case of applying the model feed solution, the difference of RED stack performance between current-voltage (I–V) and CC was extremely low, while it became higher when using natural feed solutions. This was because of more polarization and fouling that occurred because of multivalent ions and natural organic materials, respectively.

The SW desalination plant can produce a significant amount of RO brine of 60,000 m^3^ over 24 h. Therefore, 437 kW/day net power can be generated using an RO brine/RW feed solution. A 900 m^2^ solar panel with an efficiency of 18.7% would be necessary to produce this amount of energy in 24 h of stable production, which is not possible.

## Figures and Tables

**Table 1:** Ion composition of available solutions in the water desalination unit.

Solution	Conductivity [mS/cm]	Na^+^	K^+^	Mg^2+^	Ca^2+^	Cl^−^	SO_4_^2−^
RW [mmol/dm^3^]	0.34 ± 0.05	0.89	0.20	0.19	0.93	1.00	0.20
SW [mmol/dm^3^]	51.9 ± 1	469	33.0	61.0	12.0	489	25.0
RO brine [mmol/dm^3^]	81.9 ± 1	778	12.0	87.0	20.0	990	44.0

**Table 2:** Properties of ASTOM’s ion exchange membranes applied in RED stack.

Membrane	Type	IEC * [meq/g]	Water Content [-]	Resistance [Ω·cm^2^]	Thickness [µm]
CIMS	One side cation monovalent selective	2.3	0.30	2.49	150
ACS-8T	One side anion monovalent selective	1.9	0.39	2.41	150

* Ion Exchange Capacity.

**Figure A1:**
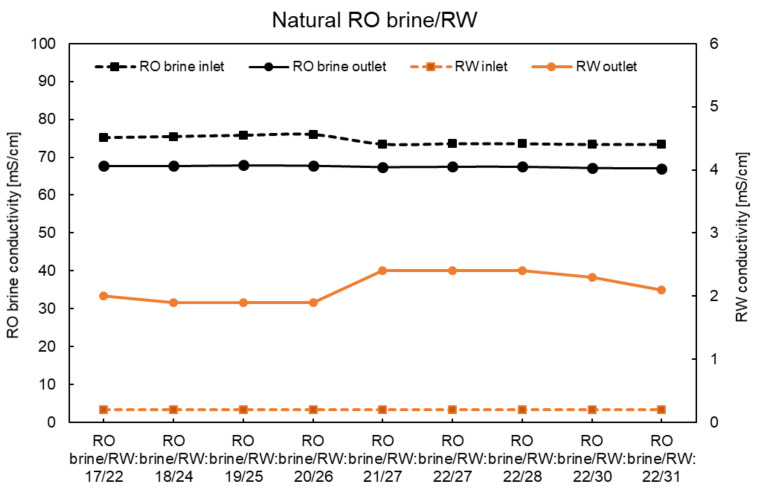
Conductivity of inlet and outlet solutions using the natural RO brine/RW at zero current.

**Figure A2:**
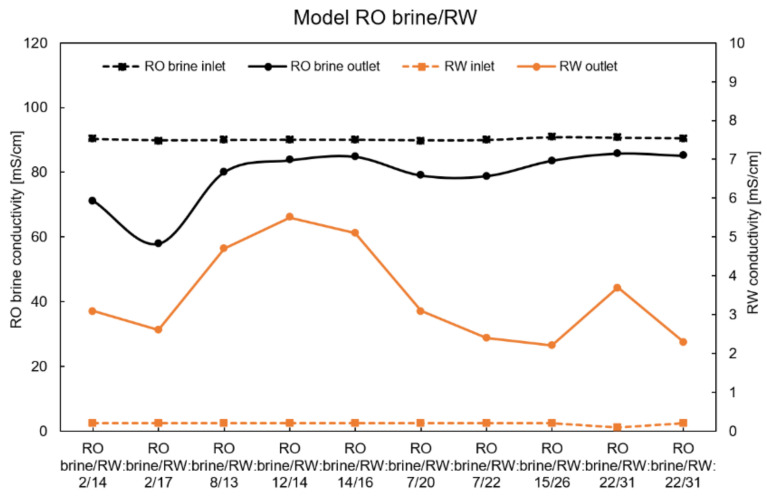
Conductivity of inlet and outlet solutions using the model RO brine/RW at zero current.

**Figure A3:**
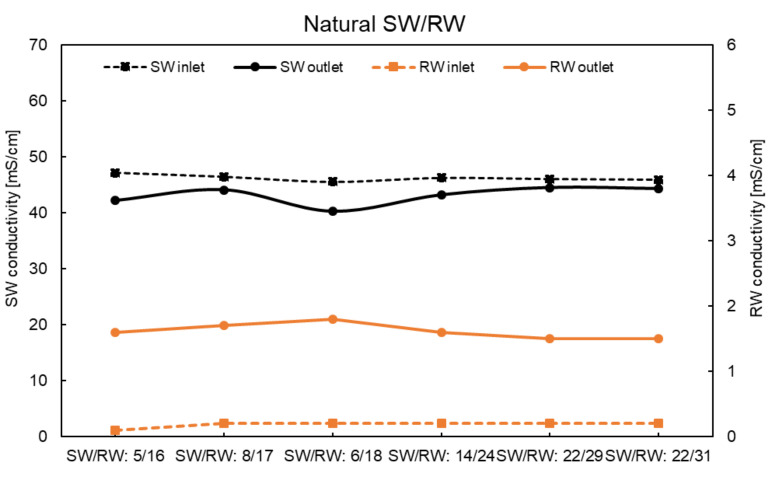
Conductivity of inlet and outlet solutions using the natural SW/RW at zero current.

**Figure A4:**
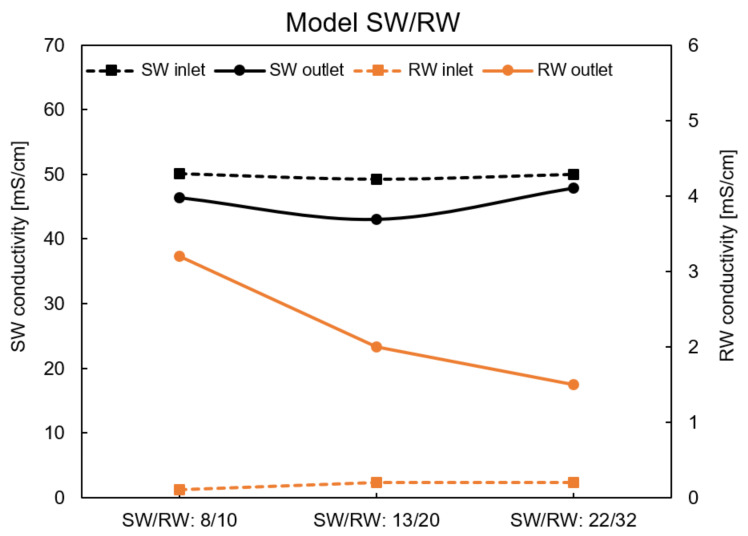
Conductivity of inlet and outlet solutions using the natural RO brine/RW at zero current.

**Figure A5:**
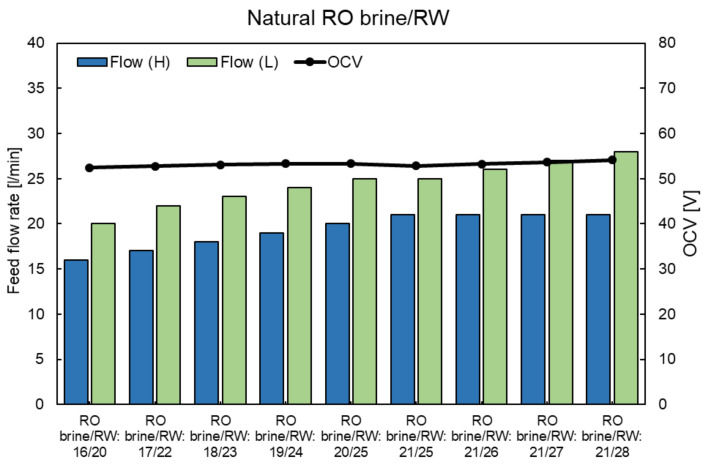
OCVs of RED stack using the natural RO brine and RW feed solution at different feed flow rates.

**Figure A6:**
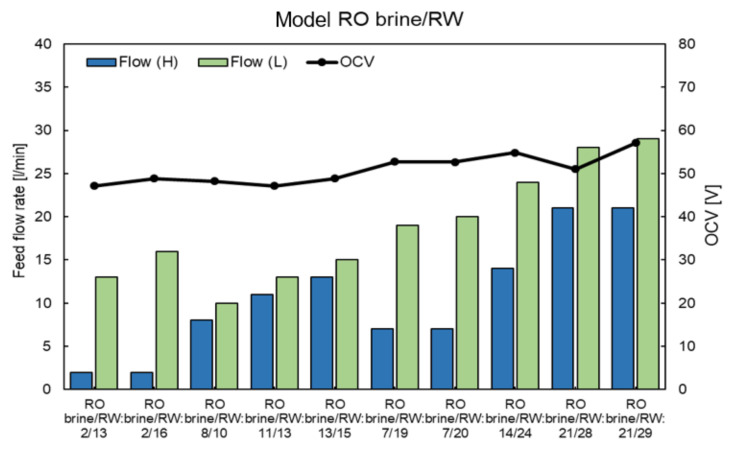
OCVs of RED stack using the model RO brine and RW feed solution at different feed flow rates.

**Figure A7:**
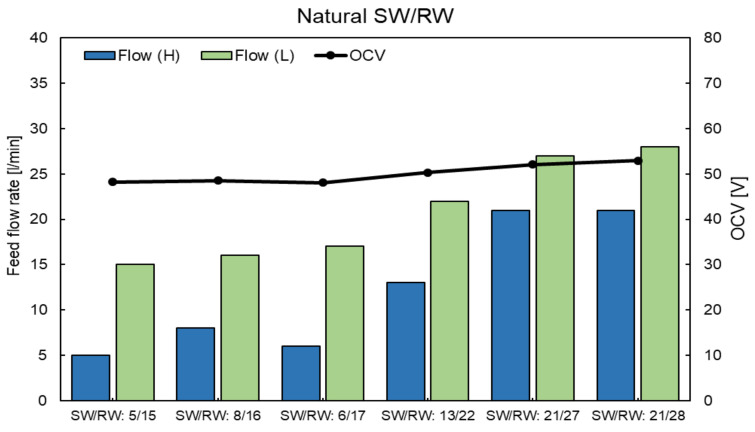
OCVs of RED stack using natural SW and RW feed solutions at different feed flow rates.

**Figure A8:**
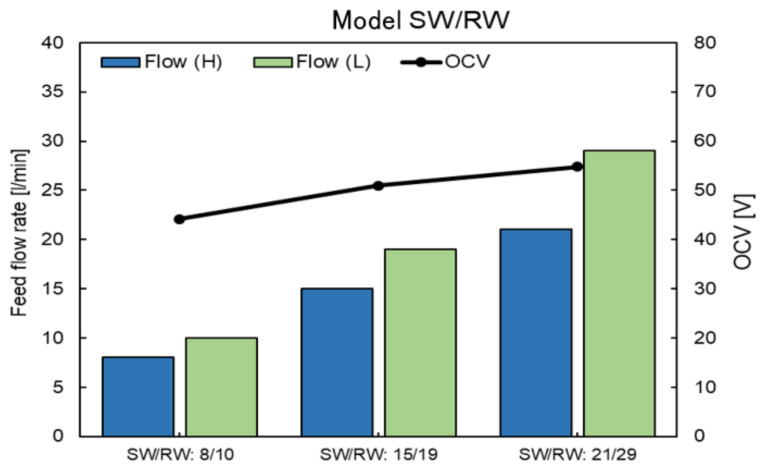
OCVs of RED stack using model SW and RW feed solutions at different feed flow rates.

**Figure A9:**
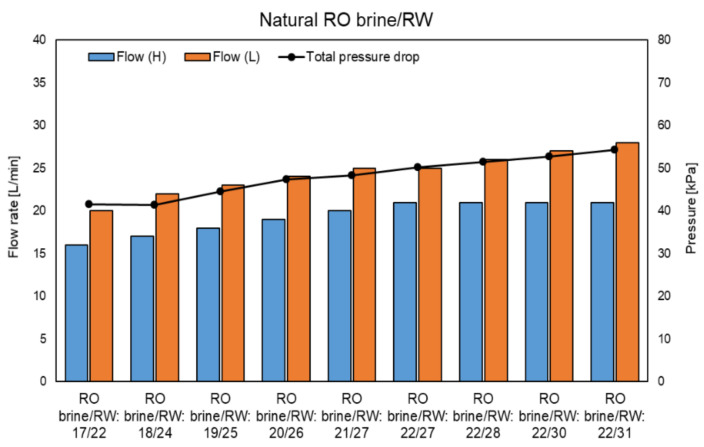
Pressure drops of RED stack as a function of the feed flow rate using the natural RO brine/RW.

**Figure A10:**
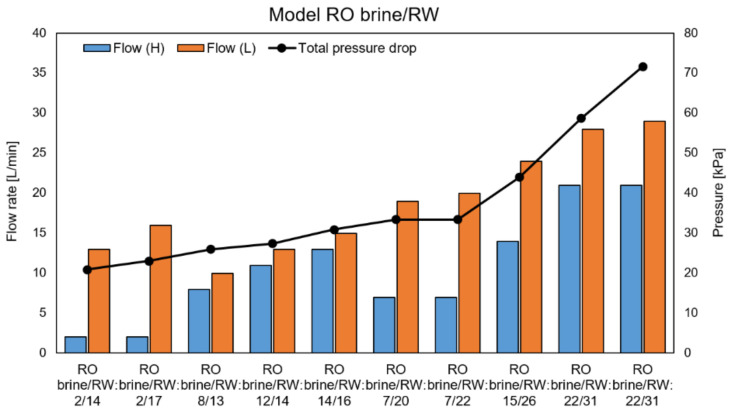
Pressure drops of RED stack as a function of the feed flow rate using model RO brine/RW.

**Figure A11:**
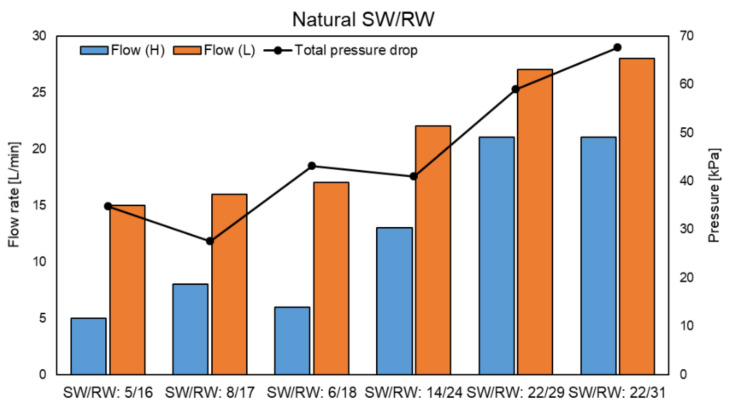
RED stack pressure drops as a function of the feed flow rate using natural SW/RW.

**Figure A12:**
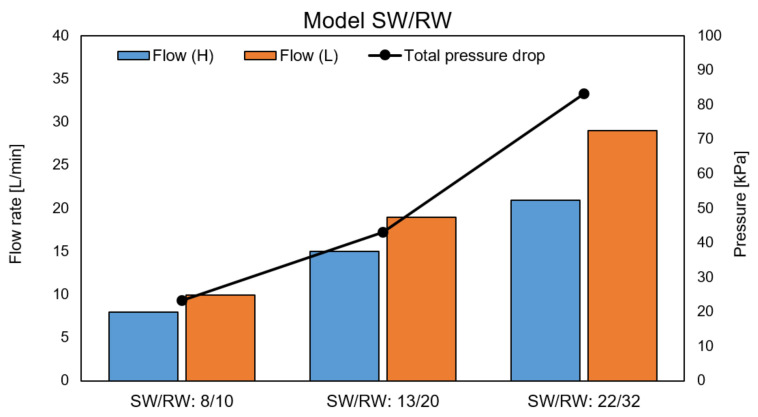
The RED stack pressure drops as a function of the feed flow rate using the model SW/RW.

## Data Availability

All data generated or analysed during this study are included in this published article.

## Appendix A

### Appendix A.1. Ion Exchange Capacity (IEC) and Water Content

The transport properties of IEMs depend on the amount and species of ion exchange groups; hence, ion exchange capacity (IEC), defined as milliequivalent per gram of a dry membrane (meq/g-dryIEM), is an important characteristic of the IEMs. Before measuring IEC, an IEM was immersed in 1.0 mol/dm^3^ KCl solution for 3 h. Then, the IEM was rinsed with deionized water to remove the non-exchange KCl electrolyte that was absorbed by the IEM. Finally, the respective IEM was immersed in 1.0 mol/dm^3^ of NaNO_3_ with the volume of 50 cm^3^ under stirring for 12 h to achieve the complete exchange of Cl^−^ in the IEM with NO_3_^−^ ions in the solution. To measure the concentration of Cl^−^ ions in the solution, C_Cl_, analysis was conducted using ion chromatography (Dionex ICS-1500). The sample membrane was immersed in 0.5 M NaCl for 24 h, and measured the wet weight, *W_w._* Then, the dried weight, *W_d_*, of the IEM was measured after keeping it in vacuum for 24 h. The water content (=*W_w_/W_d_*) was calculated from the weights. The following equation was applied to calculate the IEC of the IEM:

(A1) $$IEC=\frac{50\;\times C_{Cl}}{W_{d}}$$

### Appendix A.2. Membrane Resistance (Ω)

Membranes and the solution resistance were measured using a handmade acrylic cell consisting of two parts separated by a membrane with an effective area of 1 cm^2^, as described in our previous study [4]. The sample solution was prepared using NaCl (model seawater) with a conductivity of 49 mS/cm at 25 °C. Briefly, the sample solution was purged inside the cell and the cell was then immersed in a water bath at 20 ± 1 °C to measure the solution bulk resistance without a membrane, *R_bulk_*. In addition, the ion conductivity of the solution was measured using a conductivity meter (ES-51, HORIBA. Ltd. Kyoto, Japan). Subsequently, the same procedure was performed in the presence of a sample membrane to measure the resistance, including both the solution and membrane resistance, *R_bulk+mem_*, at a particular temperature. An alternating current (AC) of 10 kHz frequency was applied to prevent an increase in the membrane resistance via the concentration polarization effect. The membrane resistance, *R_mem_*, was then calculated from the difference between *R_bulk_* and *R_bulk+mem_* as follows:

*R_mem_* = *R_(bulk+mem)_* − *R_bulk_* (A2)

## Appendix B

The ion activity coefficient of the NaCl ($\gamma_{NaCl}$) solution at different concentrations was calculated using the following equations [36]:

(i) 0.000 < *C_NaCl_* ≤ 0.024
  (A3) $$\gamma_{NaCl}=257.97\;C_{NaCl}^{2}-11.368\;C_{NaCl}+0.9864$$
(ii) 0.0241 < *C_NaCl_* ≤ 0.190
  (A4) $$\gamma_{NaCl}=4.4627\;C_{NaCl}^{2}-1.6864\;C_{NaCl}+0.8947$$
(iii) 0.190 < *C_NaCl_*
  (A5) $$\gamma_{NaCl}=0.2461\;C_{NaCl}^{2}-0.3472\;C_{NaCl}+0.8947$$

In addition, the equivalent conductivity of different NaCl solutions can be estimated using the following equations [36]:

(i) 0.000 < *C_NaCl_* ≤ 0.00856, 0 < *k* ≤ 1.029576
  (A6) $$k=3.9485\;C_{NaCl}^{2}-119.94\;C_{NaCl}+0.0026$$
(ii) 0.00856 < *C_NaCl_* ≤ 0.172844, 1.029576 < *k* ≤ 22.720034
  (A7) $$k=85.827\;C_{NaCl}^{2}-116.6\;C_{NaCl}+0.0113$$
(iii) 0.190 < *C_NaCl_*, 22.720034 < *k*
  (A8) $$k=-15.857\;C_{NaCl}^{2}-104.13\;C_{NaCl}-0.542$$

## Appendix C

The conductivities of the inlet and outlet solutions under all feed flow rate conditions are shown in Figure A1, Figure A2, Figure A3 and Figure A4. These data were considered to calculate the theoretical OCV and leakage.

The actual OCV of all RED tests using natural and model feed solutions at different flow rates are shown in Figure A5, Figure A6, Figure A7 and Figure A8. Generally, the OCVs showed increasing behavior by increasing the feed flow rate, as discussed.

## Appendix D

The value of the pressure drop during the RED performance measurement is shown in Figure A9, Figure A10, Figure A11 and Figure A12. The pressure values are the accumulation of the pressure drop at both the high- and low-concentrate compartments. Pressure drop was used to calculate the net power value of the RED stack, as previously discussed.
